# Regular Board of Examiners

**Published:** 1898-11

**Authors:** 


### REGULAR BOARD OF EXAMINERS.

ATLANTA, GA., OCTOBER 11, 1898.

MATERIA MEDICA AND THERAPEUTICS.—BY DR. W. A. O'DANIEL.

1. Give the differential diagnosis between poisoning by iodoform and poisoning by iodine and iodides, and the treatment for iodoform poisoning, and how does iodoform poisoning usually occur?

2. Describe chronic lead poisoning, and give treatment for same.

3. Give the respective dangers to be anticipated in the administration of chloroform and of ether.

4. Name the most important soporifics, also the dose and physiological action of each.

5. What is the dose of the tincture of aconite, and its therapeutics?

6. Give the physiological action of belladonna, and the dose of its important and official preparations.

CHEMISTRY.—DR. E. R. ANTHONY.

1. Name a univalent, a bivalent, a trivalent and a quadrivalent element.

2. What is a radical?

3. Write an equation illustrating direct decomposition. Double decomposition.

4. Give me the formula of sodium chlorid. What important function does it perform in the body?

5. What is the reaction of normal urine? To what is this reaction due?

PHYSIOLOGY.—DR. E. R. ANTHONY.

1. Name the five digestive juices and briefly give me the function of each.

2. Name the valves of the heart, describe them and tell what orifice each one guards.

3. About how much air is taken into the lungs in an ordinary inspiration? How much of this is nitrogen and what is its function?

4. Name the various excretions of the body.

5. What nerve center presides over all the essential reflex acts of life (digestion, respiration, circulation)?

SURGERY.—DR. F. M. RIDLEY.

1. (a) What is a fracture? (b) How many kinds? (c) Describe each. Differential diagnosis between fracture and dislocation. Give approved treatment of fracture of upper third of femur. Of patella.

2. Give symptoms of cerebral concussion, and of cerebral compression. Differential diagnosis.

3. Give muscular guide for the ligation of femoral artery, middle third. Of ant. tibial, post tibial. Of radial.

4. Give approved treatment of gunshot wound of chest (penetrating.) Give approved treatment of gunshot wound of abdomen.

5. Differential diagnosis between chancre and chancroid? Give approved treatment of each.

ANATOMY.—DR. F. M. RIDLEY.

1. Describe elbow- and hip-joints and name possible dislocation of each.

2. Name muscles forming quadriceps extensor femoris and give origin, insertion and innervation of each.

3. Name bones forming orbital cavity and give their articulations.

4. Give relations of external carotid artery and name its branches.

5. Give origin and distribution of brachial plexus.

GYNECOLOGY.—DR. J. B. S. HOLMES.

1. What is the most frequent cause of metrorrhagia after the menopause, and how would you treat it?

2. Describe prolapsus urethræ, state what it might be mistaken for and the proper treatment of it.

3. Give symptoms and treatment of acute cystitis in the female.

4. Define membranous dysmenorrhea and give prognosis and treatment.

5. What is vicarious menstruation and how should it be treated?

OBSTETRICS.—DR. J. B. S. HOLMES.

1. Give symptoms and treatment of phlegmasia alba dolens.

2. When, how and in what doses would you use ergot in obstetrical practice?

3. State the most frequent cause of mastitis, its prevention and treatment.

4. What do you understand by inversion of the uterus and how would you treat it?

5. What are the indications for the induction of abortion and what are the best methods of inducing it?

PRACTICE.—DR. A. A. SMITH.

1. Give symptoms of urticaria, also appropriate treatment for same.

2. Give symptoms and treatment of cholera infantum.

3. What are the differential diagnostic features in spasmodic and membranous croup?

4. Give symptoms and treatment of acute dysentery.

5. Give differential diagnostic symptoms in the eruption of scarlet fever and measles.

6. Give prominent symptoms and treatment for diphtheria.

7. Give three or more of the characteristic symptoms of typhoid fever.

8. Give period of incubation in measles.

